# Nonalcoholic fatty liver disease, liver fibrosis, and structural brain imaging: The Cross‐Cohort Collaboration

**DOI:** 10.1111/ene.16048

**Published:** 2023-08-28

**Authors:** Galit Weinstein, Adrienne O'Donnell, Stefan Frenzel, Tian Xiao, Amber Yaqub, Pinar Yilmaz, Robert J. de Knegt, Gladys E. Maestre, Debora Melo van Lent, Michelle Long, Monica Gireud‐Goss, Till Ittermann, Fabian Frost, Robin Bülow, Ramachandran S. Vasan, Hans J. Grabe, M. Arfan Ikram, Alexa S. Beiser, Sudha Seshadri

**Affiliations:** ^1^ School of Public Health University of Haifa Haifa Israel; ^2^ Department of Biostatistics Boston University School of Public Health Boston Massachusetts USA; ^3^ Framingham Study Framingham Massachusetts USA; ^4^ Department of Psychiatry and Psychotherapy University Medicine Greifswald Greifswald Germany; ^5^ Department of Epidemiology Erasmus University Medical Center Rotterdam the Netherlands; ^6^ Department of Radiology & Nuclear Medicine Erasmus University Medical Center Rotterdam the Netherlands; ^7^ Department of Gastroenterology and Hepatology Erasmus University Medical Center Rotterdam the Netherlands; ^8^ Neurosciences Laboratory, Biological Research Institute and Research Institute of Cardiovascular Diseases, Faculty of Medicine Universidad del Zulia Maracaibo Venezuela Maracaibo Venezuela; ^9^ Division of Neurosciences, Department of Biomedical Sciences University of Texas Rio Grande Valley School of Medicine Edinburg Texas USA; ^10^ Glenn Biggs Institute for Alzheimer's and Neurodegenerative Diseases University of Texas Health Sciences Center San Antonio Texas USA; ^11^ Department of Neurology Boston University School of Medicine Boston Massachusetts USA; ^12^ Section of Gastroenterology, Boston Medical Center Boston University School of Medicine Boston Massachusetts USA; ^13^ Institute for Community Medicine University Medicine Greifswald Greifswald Germany; ^14^ Department of Medicine A University Medicine Greifswald Greifswald Germany; ^15^ Institute for Diagnostic Radiology and Neuroradiology University Medicine Greifswald Greifswald Germany; ^16^ Section of Preventive Medicine and Epidemiology, Department of Medicine Boston University School of Medicine Boston Massachusetts USA; ^17^ Department of Epidemiology Boston University School of Public Health Boston Massachusetts USA; ^18^ German Center for Neurodegenerative Disease, partner site Rostock/Greifswald Rostock Germany

**Keywords:** brain aging, brain MRI, liver fibrosis, nonalcoholic fatty liver disease, observational study

## Abstract

**Background and purpose:**

Prior studies reported conflicting findings regarding the association of nonalcoholic fatty liver disease (NAFLD) and liver fibrosis with measures of brain health. We examined whether NAFLD and liver fibrosis are associated with structural brain imaging measures in middle‐ and old‐age adults.

**Methods:**

In this cross‐sectional study among dementia‐ and stroke‐free individuals, data were pooled from the Offspring and Third Generation cohorts of the Framingham Heart Study (FHS), the Rotterdam Study (RS), and the Study of Health in Pomerania. NAFLD was assessed through abdominal imaging. Transient hepatic elastography (FibroScan) was used to assess liver fibrosis in FHS and RS. Linear regression models were used to explore the relation of NAFLD and liver fibrosis with brain volumes, including total brain, gray matter, hippocampus, and white matter hyperintensities, adjusting for potential confounders. Results were combined using fixed effects meta‐analysis.

**Results:**

In total, 5660 and 3022 individuals were included for NAFLD and liver fibrosis analyses, respectively. NAFLD was associated with smaller volumes of total brain (β = −3.5, 95% confidence interval [CI] = −5.4 to −1.7), total gray matter (β = −1.9, 95% CI = −3.4 to −0.3), and total cortical gray matter (β = −1.9, 95% CI = −3.7 to −0.01). In addition, liver fibrosis (defined as liver stiffness measure ≥8.2 kPa) was related to smaller total brain volumes (β = −7.3, 95% CI = −11.1 to −3.5). Heterogeneity between studies was low.

**Conclusions:**

NAFLD and liver fibrosis may be directly related to brain aging. Larger and prospective studies are warranted to validate these findings and identify liver‐related preventive strategies for neurodegeneration.

## INTRODUCTION

Nonalcoholic fatty liver disease (NAFLD) has become the most common chronic liver disease, with estimated prevalence of 25% in the general population, 60% in individuals with type 2 diabetes, and 80% in individuals with obesity [1]. The increased rates of morbidity and mortality in individuals with NAFLD stem not only from pathologies of the liver itself, but also from the multiple extrahepatic complications of NAFLD, such as type 2 diabetes and cardiovascular diseases [1].

The fact that NAFLD shares risk factors and underlying mechanisms with brain damage has raised a growing interest in the liver–brain axis [2]. Yet, the associations of NAFLD with risk of dementia [3, 4] and cognitive function [5] remain inconsistent. In the Offspring Generation of the Framingham Heart Study (FHS), NAFLD was associated with lower total brain volume independent of cardiometabolic factors including diabetes, cardiovascular diseases, and visceral adiposity [6]. However, only a few population‐based studies investigated the association of NAFLD with comprehensive brain magnetic resonance imaging (MRI) measures of neurodegeneration and vascular injury [7, 8], and defined NAFLD using blood‐based indices or self‐report [8].

The clinical and pathophysiological heterogeneity of NAFLD may partly explain the inconsistencies in previous findings linking NAFLD to various outcomes of brain health. Of note, NAFLD may include isolated steatosis but also nonalcoholic steatohepatitis (NASH), which is characterized by lobular inflammation and hepatocyte injury on histology [9]. In turn, patients with NASH are prone to disease progression to various stages of fibrosis, as well as cirrhosis and eventually hepatocellular carcinoma [9]. Mounting evidence demonstrates an association of liver fibrosis with an increased risk of stroke [10], poor cognitive function [11, 12, 13], and higher rates of incident dementia [14]. Furthermore, liver fibrosis was observed to be associated with lower total brain and hippocampal volumes in a large sample of the UK Biobank study [12]. Nevertheless, despite being cheap, noninvasive, and highly accessible [15], the use of blood‐based algorithms for detecting liver fibrosis only limits the detection of liver fibrosis, and could be improved by imaging measures such as ultrasound and elastography of the liver to increase diagnosis accuracy and robustness of previous findings [15].

Liver stiffness was assessed in the current study using vibration‐controlled transient elastography (VCTE), which is a widely used noninvasive test that provides a liver stiffness measurement through the degree of mechanically generated shear wave across the liver [16]. VCTE has excellent diagnostic accuracy and interuser reliability for detection of fibrosis [17]. Mounting evidence suggests that high liver stiffness is a predictor of mortality [18] and cardiovascular events [19]. However, the relationship between liver stiffness and measures of brain aging, to our knowledge, has not been investigated.

In this study, we aimed to investigate the cross‐sectional associations of NAFLD and liver fibrosis with MRI measures of brain aging by pooling data from three population‐based cohort studies: FHS (Offspring and Third Generation participants), the Rotterdam Study (RS), and the Study of Health in Pomerania (SHIP). We hypothesized that both NAFLD and liver fibrosis are associated with alteration in brain structure.

## METHODS

### Study sample

The study sample is based on participants from the Offspring (FHS‐Offspring) [20] and Third Generation (FHS‐3rd Gen) [21] cohorts of FHS, RS [22], and SHIP [23], all of which are part of the neurology working group of the Cross‐Cohort Collaboration Consortium. General information on the participating cohorts can be found in Appendix S1. The analytic samples from each cohort included individuals who had information from the imaging examination of NAFLD (after the age of 45 years) or liver stiffness (Fibroscan), and additionally had information on a subsequent brain MRI. NAFLD was assessed during 2011–2014 in FHS‐Offspring, 2008–2011 in FHS‐3rd Gen, 2009–2014 in RS, and 2008–2012 in SHIP. Liver stiffness was assessed during 2016–2019 in FHS‐3rd Gen and 2009–2014 in RS. Participants with prevalent stroke or dementia at the time of brain MRI examination were excluded. We also excluded participants with excessive alcohol consumption, use of steatogenic medications (i.e., didanosine, stavudine, perhexiline maleate, diethylaminoethoxyhexestrol, tamoxifen, methotrexate, 5‐fluorouracil, irinotecan, amiodarone, corticosteroids, and antiepileptic drugs [24]), and viral hepatitis. For details, refer to Appendix S1.

### Standard protocol approvals, registrations, and patient consents

Written informed consent was provided by all study participants. The study protocols were approved by the institutional review boards of the respective participating institutions (Appendix S1).

### Assessment of NAFLD and liver fibrosis

NAFLD was assessed using multidetector computed tomography (CT) in FHS. In RS and SHIP, NAFLD was defined based on abdominal Hitachi HI VISION 900 ultrasound. Only FHS and RS had measurements of liver stiffness using VCTE (Fibroscan; Echosens), which were performed by a certified operator to obtain measurements of liver fibrosis (liver stiffness measurement [LSM]).

Additional details on NAFLD and liver fibrosis ascertainment can be found in Appendix S1. We excluded invalid LSM, defined as <10 valid data points, or interquartile range/median ratio > 30% [25]. LSM was treated as a continuous as well as a binary measure. We used a cutoff of ≥7.0 kPa as an indicator of liver fibrosis, and a more conservative cutoff (less sensitive but more specific) at ≥8.2 kPa in accordance with previous studies [26].

### Brain imaging measures

Total brain volume, total gray matter volume, total cortical gray matter volume, hippocampal volume, white matter hyperintensity volume, and total intracranial volume were calculated from T1‐weighted and fluid‐attenuated inversion recovery images via automated image‐processing pipelines in all studies. Further details on study‐specific brain MRI methods can be found in Appendix S1.

### Statistical analysis

In each cohort separately, multivariate linear regression models were used to assess the relation of NAFLD with total brain, gray matter, hippocampal, and white matter hyperintensity volumes. White matter hyperintensity volumes were log‐transformed in all cohorts to normalize the skewed distribution. There was one model for each MRI measure (dependent variable) with NAFLD as the independent variable of interest. Models were adjusted for potential confounders, namely age, age‐squared, sex, total intracranial volume, and years between abdominal CT and brain MRI (Model 1). In a subsequent model (Model 2), we also adjusted for body fat (visceral adipose tissue in FHS or body mass index in RS and SHIP), prevalent hypertension, and prevalent diabetes. The associations of liver fibrosis with brain MRI measures were assessed using a similar set of models. Models from SHIP were also adjusted for study cohort (SHIP‐START and SHIP‐TREND).

Statistical analyses were conducted using SAS version 9.4, R version 4.1.0, and R version 4.0 in FHS, RS, and SHIP, respectively.

We hypothesized that there is a common effect of liver disease on brain MRI measures in all cohorts. Therefore, we used a fixed effects meta‐analysis approach to pool the cohort‐specific results. This method is also appropriate due to the small number of studies and because analyses in each participating cohort were conducted using a shared protocol [27]. However, we reran the analyses also using the random effects approach. Meta‐analysis was conducted using the inverse variance method in Review Manager version 5.4.1.

## RESULTS

Characteristics of the study samples are described in Table 1. Overall, 5660 participants from FHS‐Offspring, FHS‐3rd Gen, RS, and SHIP were included in the NAFLD and brain analyses. The cohort‐specific mean ages ranged from 53 ± 5 years in FHS‐3rd Gen to 69 ± 9 years in RS. NAFLD prevalence was 28.5% in the FHS‐Offspring cohort, 24.4% in FHS‐3rd Gen, 35.4% in RS, and 33.7% in SHIP. Liver fibrosis was only available in FHS‐3rd Gen and RS. In a total sample of 3022 participants, liver fibrosis (LSM ≥ 8.2) was present in 9.6% and 4.9% of FHS‐3rd Gen and RS participants, respectively, and liver fibrosis (LSM ≥7.0) was present in 18.4% and 9.5% of FHS‐3rd Gen and RS participants, respectively.

**TABLE 1:** Baseline characteristics by cohort.

	NAFLD				Liver fibrosis	
	FHS‐Offspring	FHS‐3rd Gen	RS	SHIP	FHS‐3rd Gen	RS
Characteristics						
*n*	463	577	2826	1794	893	2129
Time between liver measurement and brain MRI, years	3.67 ± 1.21	1.25 ± 1.00	0.1 ± 0.2	0.07 ± 0.17	0.05 ± 1.09	0.1 ± 0.2
Age, years	68.1 ± 8.2	53.0 ± 5.3	69.3 ± 8.9	59.9 ± 9.7	54.7 ± 8.4	67.4 ± 8.4
Male	221 (47.7)	304 (52.7)	1201 (42.5)	811 (45.2)	426 (47.7)	952 (44.7)
Caucasian	458 (98.9)	577 (100.0)	2414 (96.6)	NA	887 (99.3)	1800 (96.3)
APOE ɛ4+	109 (24.1)	126 (22.9)	701 (24.8)	389 (22.9)	198 (23.1)	549 (25.8)
Visceral adipose tissue	2545.8 ± 1428.9	2207.9 ± 1400.0	NA	NA	NA	NA
Body mass index, kg/m^2^	28.2 ± 4.8	28.2 ± 5.4	27.3 ± 4.1	28.3 ± 4.4	28.1 ± 5.2	27.0 ± 3.8
Diabetes	59 (13.1)	27 (4.7)	411 (14.5)	201 (11.2)	61 (6.9)	284 (13.3)
Hypertension	247 (53.4)	151 (26.3)	2025 (71.7)	1018 (56.8)	267 (30.0)	1431 (67.2)
Exposures						
NAFLD	132 (28.5)	141 (24.4)	1000 (35.4)	605 (33.7)	NA	NA
Liver fibrosis, continuous LSM	NA	NA	NA	NA	5.8 ± 4.2	5.1 ± 2.1
Liver fibrosis, LSM ≥ 8.2 kPa	NA	NA	NA	NA	86 (9.6)	104 (4.9)
Liver fibrosis, LSM ≥ 7.0 kPa	NA	NA	NA	NA	164 (18.4)	202 (9.5)
Outcomes						
Total brain volume, mL	959.7 ± 100.4	1013.2 ± 105.4	922.6 ± 97.3	977.5 ± 100.2	1005.6 ± 103.4	933.4 ± 97.1
Total gray matter volume, mL	516.7 ± 52.1	544.6 ± 53.5	524.6 ± 54.2	502 ± 48.5	552.7 ± 51.5	529.6 ± 54.4
Total cortical gray matter volume, mL	474.8 ± 48.7	503.2 ± 50.4	NA	448.2 ± 44.5	510.8 ± 48.7	NA
Hippocampal volume, mL	6.6 ± 0.7	6.9 ± 0.7	6.6 ± 0.9	7.7 ± 0.8	7.0 ± 0.7	6.7 ± 0.9
WMHV, mL	2.7 [1.3, 6.2]	0.6 [0.3, 1.1]	3.8 [1.9, 8.0]	2.8 [0.10, 0.90]	0.5 [0.2, 1.3]	3.3 [1.8, 6.6]
Total intracranial volume, mL	1279.0 ± 127.7	1295.0 ± 129.0	1133.0 ± 116.7	1566.5 ± 157.3	1256.0 ± 123.7	1137.3 ± 118.1

*Note*: Values are presented as mean ± SD, frequency (percent) or median [Q1, Q3].

Abbreviations: 3rd Gen, Third Generation cohort; APOE, apolipoprotein E; FHS, Framingham Heart Study; LSM, liver stiffness measure; MRI, magnetic resonance imaging; NA, not available; NAFLD, nonalcoholic fatty liver disease; RS, Rotterdam Study; SHIP, Study of Health in Pomerania; WMHV, White matter hyperintensity volume.

### Nonalcoholic fatty liver disease

**FIGURE 1:**
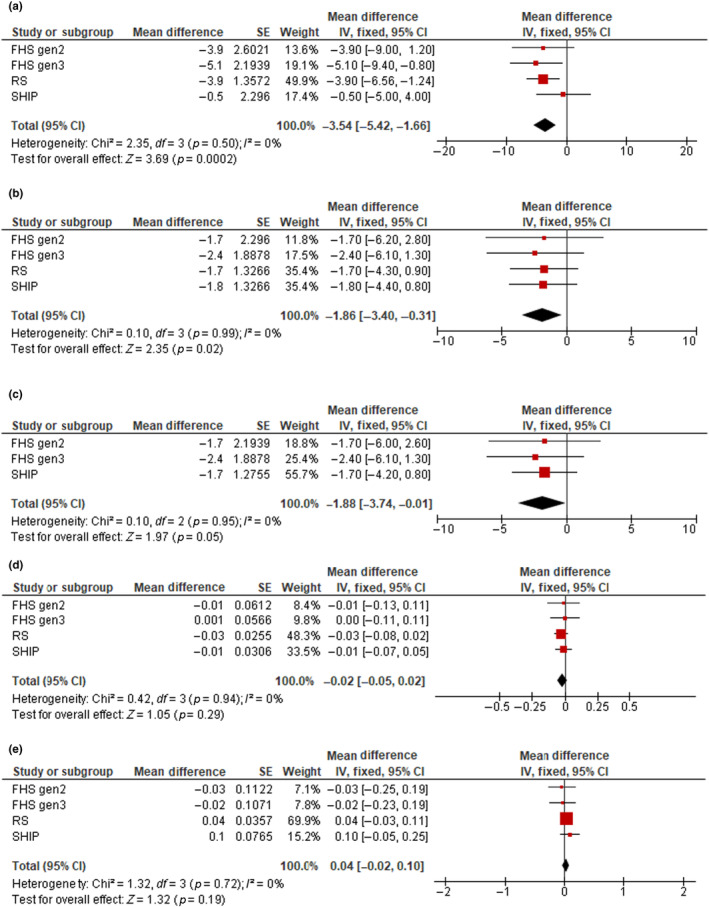
Forest plots of the associations between nonalcoholic fatty liver disease (NAFLD) and total brain volume (a), total gray matter volume (b), total cortical gray matter volume (c), hippocampal volume (d), and white matter hyperintensity volume (e). Cohort‐specific effect sizes (red squares) represent mean differences of each brain magnetic resonance imaging (MRI) outcome comparing clinically significant liver fibrosis to no fibrosis. Horizontal lines extending from squares represent 95% confidence intervals (CIs). Pooled estimates (black diamonds) were combined using fixed effects meta‐analysis. Models adjust for age, age‐squared, sex, total intracranial volume, ethnicity, time between NAFLD assessment and MRI, body fat, hypertension, and diabetes. FHS, Framingham Heart Study; IV, intravenous; RS, Rotterdam Study; SHIP, Study of Health in Pomerania.

Cohort‐specific associations of NAFLD and liver fibrosis with brain MRI measures are shown in Table S1. Pooled estimates of the associations between NAFLD and brain MRI measures are presented in Table 2 and Figure 1. In the basic model, the presence of NAFLD was associated with lower volumes of total brain (mean difference = −5.1, 95% confidence interval [CI] = −6.8 to −3.4, *p* < 0.001), total gray matter (mean difference = −2.2, 95% CI = −3.6 to −0.8, *p* = 0.003), and total cortical gray matter (mean difference = −3.1, 95% CI = −4.8 to −1.4, *p* < 0.001), and with higher white matter hyperintensity volume (mean difference = 0.07, 95% CI = 0.02–0.12, *p* = 0.010). The associations with total brain, total gray matter, and total cortical gray matter volumes attenuated but remained statistically significant after additional adjustments for body fat, prevalent hypertension, and prevalent diabetes (mean difference = −3.5, 95% CI = −5.4 to −1.7, *p* < 0.001 for total brain; mean difference = −1.9, 95% CI = −3.4 to −0.3, *p* = 0.020 for total gray matter; and mean difference = −1.9, 95% CI = −3.7 to −0.01, *p* = 0.05 for total cortical gray matter volumes). However, the association with white matter hyperintensity volume was no longer significant after further adjustment (mean difference = 0.04,[95% CI = −0.02 to 0.10, *p* = 0.19). No significant associations were observed between NAFLD and hippocampal volume. There was a low and nonsignificant heterogeneity between studies. Similar findings were observed when a random effects meta‐analysis approach was used (Table S2).

**TABLE 2:** Pooled estimates of the associations between NAFLD and brain MRI measures.

Outcome	Studies, *n*	Model 1					Model 2				
		Heterogeneity					Heterogeneity				
		Chi^2^	*p*	*I* ^2^ (%)	β (95% CI)	*p*	Chi^2^	*p*	*I* ^2^ (%)	β (95% CI)	*p*
Total brain volume	4	0.90	0.82	0	**−5.1 (−6.8 to −3.4)**	**<0.001**	2.35	0.50	0	**−3.5 (−5.4 to −1.7)**	**<0.001**
Total gray matter volume	4	3.22	0.36	7	**−2.2 (−3.6 to −0.8)**	**0.003**	0.10	0.99	0	**−1.9 (−3.4 to −0.3)**	**0.02**
Total cortical gray matter volume	3	0.97	0.61	0	**−3.1 (−4.8 to −1.4)**	**<0.001**	0.10	0.95	0	**−1.9 (−3.7 to −0.01)**	**0.05**
Hippocampal volume	4	1.38	0.71	0	−0.02 (−0.05 to 0.02)	0.34	0.42	0.94	0	−0.02 (−0.05 to 0.02)	0.29
WMHV	4	1.77	0.62	0	**0. 07 (0.02 to 0.12)**	**0.01**	1.32	0.72	0	0.04 (−0.02 to 0.10)	0.19

*Note*: Model 1 adjusted for age, age‐squared, sex, total intracranial volume, and time between NAFLD assessment and MRI. Model 2 additionally adjusted for body fat, prevalent hypertension, and prevalent diabetes. Values in bold indicate *p* < 0.05.

Abbreviations: CI, confidence interval; *I* ^2^, heterogeneity index; MRI, magnetic resonance imaging; NAFLD, nonalcoholic fatty liver disease; WMHV, white matter hyperintensity volume.

### Liver fibrosis

**FIGURE 2:**
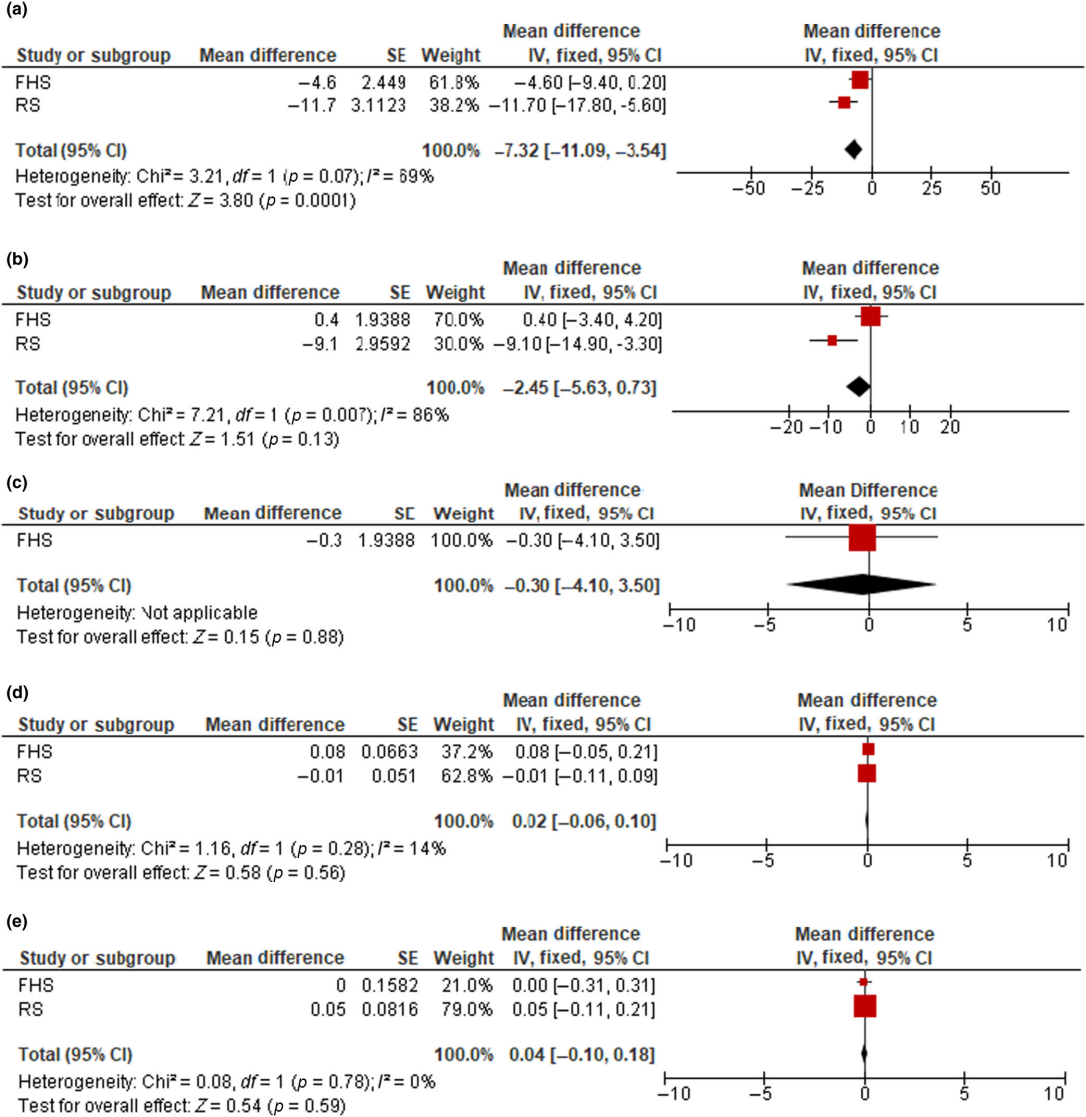
Forest plots of the associations between liver fibrosis (liver stiffness measure ≥ 8.2 kPa) and total brain volume (a), total gray matter volume (b), total cortical gray matter volume (c), hippocampal volume (d), and white matter hyperintensity volume (e). Cohort‐specific effect sizes (red squares) represent mean differences of each brain magnetic resonance imaging (MRI) outcome comparing clinically significant liver fibrosis to no fibrosis. Horizontal lines extending from squares represent 95% confidence intervals (CI). Pooled estimates (black diamonds) were combined using fixed effects meta‐analysis. Models adjust for age, age‐squared, sex, total intracranial volume, ethnicity, time between nonalcoholic fatty liver disease assessment and MRI, body mass index, hypertension, and diabetes. FHS, Framingham Heart Study; IV, intravenous; RS, Rotterdam Study.

Pooled estimates of the associations between liver stiffness measures and brain MRI measures are listed in Table 3 (Models 1 and 2). In addition, Figure 2 demonstrates the associations between liver fibrosis (using the 8.2‐kPa cutoff) and brain MRI measures in Model 2. Cohort‐specific associations between liver stiffness/fibrosis measures and brain MRI measures are listed in Table S1.

**TABLE 3:** Pooled estimates of the associations between liver fibrosis and brain MRI measures.

	Outcome	Studies, *n*	Model 1					Model 2				
			Heterogeneity					Heterogeneity				
			Chi^2^	*p*	*I* ^2^ (%)	Total effect (95% CI)	*p*	Chi^2^	*p*	*I* ^2^ (%)	Total effect (95% CI)	*p*
Liver stiffness measure, continuous	Total brain volume	2	1.60	0.21	37	**−3.9 (−6.8 to −1.1)**	**0.007**	1.57	0.21	36	−2.8 (−5.7 to 0.1)	0.06
	Total gray matter volume	2	0.57	0.45	0	**−2.4 (−4.7 to −0.11)**	**0.04**	0.93	0.33	0	−2.1 (−4.5 to 0.4)	0.09
	Total cortical gray matter volume	1	N/A	N/A	N/A	−1.6 (−4.6 to 1.4)	0.30	N/A	N/A	N/A	−1.4 (−4.4 to 1.6)	0.36
	Hippocampal volume	2	4.4	0.04	77	0.02 (−0.03 to 0.08)	0.44	2.09	0.15	52	0.02 (−0.04 to 0.08)	0.50
	WMHV	2	0.09	0.77	0	0.08 (−0.04 to 0.18)	0.13	0.01	0.94	0	0.03 (−0.07 to 0.13)	0.54
Liver fibrosis, LSM ≥ 8.2 vs. LSM < 8.2 kPa	Total brain volume	2	3.32	0.07	70	**−8.6 (−12.3 to −4.9)**	**<0.001**	3.21	0.07	69	**−7.3 (−11.1 to −3.5)**	**<0.001**
	Total gray matter volume	2	6.55	0.01	85	−2.8 (−5.9 to 1.23)	0.07	7.21	0.007	86	−2.5 (−5.6 to 0.7)	0.13
	Total cortical gray matter volume	1	N/A	N/A	N/A	−0.7 (−4.4 to 3.0)	0.71	N/A	N/A	N/A	−0.3 (−4.1 to 3.5)	0.88
	Hippocampal volume	2	2.27	0.13	56	0.03 (−0.05 to 0.11)	0.46	1.16	0.28	14	0.02 (−0.06 to 0.10)	0.56
	WMHV	2	0.05	0.82	0	0.09 (−0.04 to 0.23)	0.18	0.08	0.78	0	0.04 (−0.10 to 0.18)	0.59
Liver fibrosis, LSM ≥ 7.0 vs. LSM < 7.0 kPa	Total brain volume	2	0.97	0.32	0	**−6.3 (−9.1 to −3.5)**	**<0.001**	0.93	0.34	0	**−5.2 (−8.0 to −2.4)**	**<0.001**
	Total gray matter volume	2	0.12	0.73	0	**−2.4 (−4.7 to −0.02)**	**0.05**	0.21	0.65	0	−2.2 (−4.5 to 0.2)	0.07
	Total cortical gray matter volume	1	N/A	N/A	N/A	**−2.8 (−5.6 to 0.00)**	**0.05**	N/A	N/A	N/A	−2.7 (−5.6 to 0.2)	0.07
	Hippocampal volume	2	0.66	0.42	0	0.0 (−0.06 to 0.06)	0.95	0.12	0.73	0	−0.01 (−0.07 to 0.04)	0.66
	WMHV	2	0.48	0.49	0	**0.11 (0.01 to 0.21)**	**0.03**	0.38	0.54	0	0.06 (−0.03 to 0.16)	0.20

*Note*: Model 1 adjusted for age, age‐squared, sex, total intracranial volume, and time between fibrosis assessment and MRI. Model 2 additionally adjusted for body fat, prevalent hypertension, and prevalent diabetes. Values in bold indicate *p* < 0.05.

Abbreviations: *I* ^2^, heterogeneity index; LSM, liver stiffness measure; MRI, magnetic resonance imaging; N/A, not applicable; WMHV, white matter hyperintensity volume.

Each 1‐kPA increment in LSM was associated with lower total (mean difference = −3.9, 95% CI = −6.8 to −1.1, *p* = 0.007) and gray matter brain volumes (mean difference = −2.4, 95% CI = −4.7 to −0.11, *p* = 0.04) in the basic model. However, these associations attenuated and became nonsignificant after additional adjustments (Table 3). The presence of liver fibrosis using the 8.2‐kPa cutoff was related to lower total brain volumes in Model 1 (mean difference = −8.6, 95% CI = −12.3 to −4.9, *p* < 0.001). This association slightly attenuated but remained statistically significant after additional adjustments for body fat, prevalent hypertension, diabetes (mean difference = −7.3, 95% CI = −11.1 to −3.5, *p* < 0.001). Using the LSM cutoff of 7.0 kPa, we found that in the basic model liver fibrosis was significantly related to lower total brain, total gray and cortical gray matter volumes, and higher white matter hyperintensity volume (mean difference = −6.3, 95% CI = −9.1 to −3.5, *p* < 0.001; −2.4, 95% CI = −4.7 to −0.02, *p* = 0.05; −2.8, 95% CI = −5.6 to 0.00, *p* = 0.05; and 0.11, 95% CI = 0.01–0.21, *p* = 0.03). However, these associations attenuated and became nonsignificant after further adjustment, with the exception of total brain volume, which remained statistically significant (−5.2, 95% CI = −8.0 to −2.4, *p* < 0.001).

The results were similar when random effects rather than fixed effects meta‐analysis was done (Table S3).

## DISCUSSION

This pooled analysis from three community‐based cohorts suggests that NAFLD may be related to alterations in brain structure, such as lower total brain and gray matter volumes. In addition, based on data from two cohorts, we show, to our knowledge for the first time, that a US‐based liver stiffness measure of hepatic fibrosis (Fibroscan) is associated with imaging markers of neurodegeneration. These associations were significant even after adjustment for demographic and clinical factors, including adiposity, prevalent hypertension, and diabetes. The current research expands a previous study using data from the FHS‐Offspring cohort, which demonstrated an association between NAFLD and smaller total brain volume [6]. Here, we pooled data from several studies to increase statistical power and added liver fibrosis as an exposure.

Studies assessing the link of NAFLD with brain aging and vascular brain pathologies are sparse. In line with our findings, a previous community‐based cohort study of 351 middle‐aged and older adults demonstrated an association of increased MRI‐based hepatic fat with smaller cingulate gyrus and hippocampus gray matter volumes [7]. In contrast, a post hoc analysis of the ACCORD and SPRINT trials did not find an association between NAFLD and brain imaging markers [8]. However, these prior analyses were done among individuals with pre‐existing comorbidities, such as diabetes and hypertension. Furthermore, the prior analysis defined NAFLD based on a serum‐based index (i.e., Dallas Steatosis Index in ACCORD) and self‐reported chronic liver disease in SPRINT [8]. In contrast, we used imaging data to assess the presence of NAFLD, which are more accurate in quantifying liver fat, particularly among healthy, nonsymptomatic participants [28].

Our results suggest that increased liver fibrosis, particularly when using liver stiffness of ≥8.2 kPa, may be related to increased total brain atrophy. Emerging evidence suggests that high liver stiffness is a predictor of mortality [29] and cardiovascular events [19]. Moreover, in accordance with our findings, a recent study among ~42,000 participants of the UK Biobank demonstrated a link between liver stiffness and lower total brain volume [12]. Yet, in contrast to our results, the prior UK Biobank study also demonstrated an association of fibrosis with lower hippocampal volume [12]. The latter discrepancy may stem from differences in ascertainment of liver fibrosis, which was done indirectly through serum‐based markers in the former study. Alternatively, this discrepancy may be linked to differences in population characteristics, which may affect the prevalence of liver fibrosis (9.6% in FHS and 2.2% in UK Biobank).

Our findings, in light of previous observations, may expand current knowledge about the underlying mechanisms linking NAFLD and brain health. Interestingly, we did not observe smaller hippocampal volumes in individuals with NAFLD or liver fibrosis. Despite substantial evidence showing that atrophy of the hippocampus is the first sign of a neurodegenerative process in Alzheimer disease (AD), more recent evidence suggests that brain volume loss originates in the gray matter, particularly in the thalamus, rather than the hippocampus [30]. That total and gray matter volumes were reduced in participants with NAFLD could imply that NAFLD may be associated with age‐related, non‐AD brain atrophy [31]. Yet, a previous study among participants of FHS demonstrated increased brain amyloid‐β deposition and tau pathology, which are direct AD markers, in relation to NAFLD [32]. Furthermore, altered liver enzymes, which are often observed in NAFLD, were related to increased amyloid‐β deposition as well as brain atrophy and other AD endophenotypes in the AD Neuroimaging Initiative cohort [33]. Thus, mechanisms such as neuroinflammation, inappropriate amyloid‐β clearance, or toxic metabolites produced by the injured liver could lead to AD pathology [34], which in turn may manifest as total brain and gray matter loss [35].

We found no statistically significant associations of NAFLD and liver fibrosis with white matter hyperintensity volume. This observation is supported by a recent UK Biobank study [12], as well as by another study among 300 community‐dwelling participants that found no significant associations between liver fibrosis assessed by VCTE and white matter hyperintensities [36]. Yet, other studies observed positive associations between NAFLD, particularly with advanced liver fibrosis, and white matter hyperintensities [37, 38], or observed unexpected negative associations [39]. The conflicting findings may be partly explained by the difference in study population, which in the former studies was comprised of adults who underwent a health screening examination [38, 39] or NAFLD patients recruited from a gastrointestinal and liver unit [37]. Although white matter hyperintensity volume may reflect nonvascular mechanisms, including neurodegeneration caused by AD pathology, it is also a marker of cerebral small‐vessel disease (CSVD) [40]. Thus, the lack of association with white matter hyperintensity volume in our study may stand in contrast to a large body of evidence showing associations of NAFLD with CSVD burden manifested by lacunes, microbleeds [38], and other markers of subclinical vascular dysfunction [41]. Yet, prior evidence also indicates that white matter hyperintensities are seen mostly during advanced liver disease stages (i.e., liver cirrhosis) where they reflect hepatic encephalopathy rather than CSVD [42]. Taken together, our findings among community‐dwelling adults may highlight early changes of brain aging compatible with the relatively young age and healthy samples of participants with no overt liver disease. These observations are in line with preclinical data that highlight the possible role of metabolic imbalance, present even during the early stages of NAFLD progression, in structural damage to brain cells and neurodegeneration [43]. Furthermore, neurodegeneration can result from hepatic dysfunction, which impedes amyloid‐β clearance and promotes generation of toxic sphingolipids, including ceramides and sphingomyelin [44].

Strengths of the current study include a large sample size, the coordinated meta‐analytic approach, and the inclusion of well‐characterized cohorts with rich information on metabolic and lifestyle covariates. Of note, both NAFLD and liver fibrosis were assessed using validated, imaging‐based measures that show superiority over serum‐based indices [45]. In particular, VCTE has increased validity compared to serum‐based biomarkers [46], and has been recommended by the European Association for the Study of the Liver as a noninvasive standard for the measurement of liver fibrosis [47]. We also acknowledge several limitations. First, due to the cross‐sectional study design, we cannot infer temporal relations between liver disease and neurodegenerative and vascular brain imaging markers. Second, although we adjusted for important potential confounders, we cannot rule out the possibility of residual confounding (e.g., genetic factors or additional clinical measurements). Lastly, the external validity of our findings may be limited, because our samples are predominantly of European origin, with relatively high socioeconomic status.

In conclusion, results of the current study provide further support for the possible association between liver disease and brain aging, in predominantly healthy older adults. Although NAFLD and liver fibrosis pose a growing burden on the population [1], awareness for these conditions among patients and physicians remains low [48, 49]. Future investigations are needed to explore whether early identification and treatment for NAFLD may improve brain health.

## AUTHOR CONTRIBUTIONS

**Galit Weinstein**: Conceptualization; writing–original draft; investigation; methodology; formal analysis. **Adrienne O'Donnell**: Writing–review & editing; formal analysis; conceptualization; methodology. **Stefan Frenzel**: Conceptualization; writing–review & editing; methodology; formal analysis. **Tian Xiao**: Writing–review & editing; conceptualization; formal analysis; methodology. **Amber Yaqub**: Conceptualization; writing–review & editing; formal analysis; methodology. **Pinar Yilmaz**: Data curation; conceptualization; writing–review & editing; methodology. **Robert J. de Knegt**: Conceptualization; writing–review & editing; methodology. **Gladys E. Maestre**: Conceptualization; methodology; writing–review & editing. **Debora Melo van Lent**: Conceptualization; writing–review & editing. **Michelle Long**: Conceptualization; writing–review & editing; methodology. **Monica Gireud‐Goss**: Project administration; conceptualization. **Till Ittermann**: Conceptualization; writing–review & editing. **Fabian Frost**: Conceptualization; writing–review & editing. **Robin Bülow**: Conceptualization; writing–review & editing; data curation. **Ramachandran S. Vasan**: Conceptualization; writing–review & editing; methodology; resources; supervision. **Hans J. Grabe**: Resources; supervision; writing–review & editing; methodology; conceptualization. **M. Arfan Ikram**: Conceptualization; writing–review & editing; methodology; resources; supervision. **Alexa S. Beiser**: Conceptualization; investigation; writing–review & editing; methodology; software; formal analysis; data curation; supervision. **Sudha Seshadri**: Conceptualization; investigation; writing–review & editing; methodology; supervision; resources.

## FUNDING INFORMATION

This study was supported by grants from the National Institute on Aging (1RF1AG059421‐01 and P30 AG066546). Additionally, information on cohort‐specific funding is provided in the supplementary material.

## CONFLICT OF INTEREST STATEMENT

H.J.G. has received travel grants and speaker honoraria from Fresenius Medical Care, Neuraxpharm, Servier, and Janssen Cilag as well as research funding from Fresenius Medical Care. None of the other authors has any conflict of interest to disclose.

## Supporting information


APPENDIX S1

TABLE S1–S3

## Data Availability

The data that support the findings of this study are available on request from the corresponding author. The data are not publicly available due to privacy or ethical restrictions.
