# Supplementary material for: Nonalcoholic fatty liver disease, liver fibrosis, and structural brain imaging: The Cross-Cohort Collaboration
Source: Eur J Neurol. Author manuscript; Available in PMC 2024 Feb 5. (PMC10840827; doi:10.1111/ene.16048)
Supplement: Supplementary file 1 [file NIHMS1931502-supplement-Supplement_1_Liver.docx]

**Supplementary Table 1.** Cohort-specific associations of NAFLD and liver fibrosis with brain MRI measures

|  |  |  | Model 1 |  | Model 2 |  |
| --- | --- | --- | --- | --- | --- | --- |
| Exposure | Outcome | Cohort | N | Mean difference (95% CI) | N | Mean difference (95% CI) |
| NAFLD | TBV | FHS-Offspring | 463 | -4.5 (-9.0, 0.1) | 447 | -3.9 (-9.0, 1.2) |
|  |  | FHS-Gen 3 | 577 | **-5.7 (-9.5, -2.0)** | 571 | **-5.1 (-9.4, -0.8)** |
|  |  | RS | 2,826 | **-5.56 (-7.97, -3.15)** | 2,825 | **-3.90 (-6.56, -1.24)** |
|  |  | SHIP | 1,794 | -3.4 (-7.8, 1.1) | 1,791 | -0.5 (-5.0, 4.0) |
|  | TGV | FHS-Offspring | 463 | -1.6 (-5.6, 2.4) | 437 | -1.7 (-6.2, 2.8) |
|  |  | FHS-Gen 3 | 577 | **-3.4 (-6.6, -0.2)** | 571 | -2.4 (-6.1, 1.3) |
|  |  | RS | 2,826 | -0.73 (-3.03, 1.57) | 2,825 | -1.72 (-4.27, 0.83) |
|  |  | SHIP | 1,794 | **-3.5 (-6.1, -0.9)** | 1,791 | -1.8 (-4.4, 0.9) |
|  | TCGV | FHS-Offspring | 463 | -1.4 (-5.3, 2.5) | 437 | -1.7 (-6.0, 2.7) |
|  |  | FHS-Gen 3 | 577 | **-3.2 (-6.4, -0.02)** | 571 | -2.4 (-6.1, 1.3) |
|  |  | SHIP | 1,794 | **-3.7 (-6.1, -1.2)** | 1,791 | -1.7 (-4.3, 0.8) |
|  | HV | FHS-Offspring | 463 | 0.004 (-0.11, 0.12) | 437 | -0.01 (-0.13, 0.12) |
|  |  | FHS-Gen 3 | 577 | 0.01 (-0.09, 0.10) | 571 | 0.00 (-0.11, 0.12) |
|  |  | RS | 2,612 | -0.03 (-0.07, 0.01) | 2,611 | -0.03 (-0.08, 0.01) |
|  |  | SHIP | 1,794 | 0.01 (-0.06, 0.07) | 1,791 | -0.01 (-0.07, 0.06) |
|  | WMHV^†^ | FHS-Offspring | 463 | -0.06 (-0.27, 0.14) | 437 | -0.03 (-0.25, 0.19) |
|  |  | FHS-Gen 3 | 577 | 0.09 (-0.09, 0.28) | 571 | -0.02 (-0.23, 0.19) |
|  |  | RS | 2,826 | **0.08 (0.02, 0.15)** | 2,825 | 0.04 (-0.03, 0.11) |
|  |  | SHIP | 1,788 | 0.04 (-0.11, 0.19) | 1,785 | 0.1 (-0.05,0.25) |
| Liver stiffness measure (continuous) | TBV | FHS-Gen 3 | 893 | -2.3 (-6.1, 1.4) | 889 | -1.1 (-5.0, 2.8) |
|  |  | RS | 2129 | **-5.99 (-10.25, -1.72)** | 2,128 | **-4.81 (-9.11, -0.52)** |
|  | TGV | FHS-Gen 3 | 893 | -1.8 (-4.6, 1.1) | 889 | -1.2 (-4.2, 1.8) |
|  |  | RS | 2,129 | -3.70 (-7.75, 0.34) | 2,128 | -3.70 (-7.79, 0.38) |
|  | TCGV | FHS-Gen 3 | 893 | -1.6 (-4.6, 1.3) | 889 | -1.4 (-4.4, 1.6) |
|  | HV | FHS-Gen 3 | 893 | 0.11 (0.01, 0.21) | 889 | 0.08 (-0.02, 0.18) |
|  |  | RS | 1,963 | -0.02 (-0.09, 0.05) | 1,962 | -0.01 (-0.08, 0.06) |
|  | WMHV^†^ | FHS-Gen 3 | 893 | 0.11 (-0.13, 0.35) | 889 | 0.04 (-0.21, 0.29) |
|  |  | RS | 2,129 | 0.07 (-0.04, 0.18) | 2,128 | 0.03 (-0.08, 0.14) |
| Liver fibrosis (LSM ≥8.2 vs. <8.2 kPa) | TBV | FHS-Gen 3 | 893 | **-6.0 (-10.6, -1.3)** | 855 | -4.6 (-9.4, 0.2) |
|  |  | RS | 2,129 | **-13.10 (-19.20, -7.01)** | 2,128 | **-11.7 (-17.8, -5.6)** |
|  | TGV | FHS-Gen 3 | 893 | -0.3 (-3.9, 3.3) | 855 | 0.4 (-3.4, 4.1) |
|  |  | RS | 2,129 | **-9.06 (-14.84, -3.27)** | 2,128 | **-9.12 (-14.94, -3.29)** |
|  | TCGV | FHS-Gen 3 | 893 | -0.7 (-4.4, 3.0) | 855 | -0.3 (-4.1, 3.5) |
|  | HV | FHS-Gen 3 | 893 | 0.10 (-0.02, 0.23) | 855 | 0.08 (-0.05, 0.21) |
|  |  | RS | 1,963 | -0.02 (-0.12, 0.08) | 1,962 | -0.01 (-0.11, 0.09) |
|  | WMHV^†^ | FHS-Gen 3 | 893 | 0.06 (-0.24, 0.36) | 855 | 0.00 (-0.31, 0.31) |
|  |  | RS | 2,129 | 0.10 (-0.05, 0.26) | 2,128 | 0.05 (-0.11, 0.21) |
| Liver fibrosis (LSM ≥7.0 vs. <7.0 kPa) | TBV | FHS-Gen 3 | 893 | **-5.2 (-8.8, -1.6)** | **889** | **-4.1 (-7.7, -0.4)** |
|  |  | RS | 2,129 | **-8.1 (-12.6, -3.6)** | 2,128 | **-6.95 (-11.50, -2.40)** |
|  | TGV | FHS-Gen 3 | 893 | -2.1 (-4.9, 0.6) | 889 | -1.8 (-4.6, 1.1) |
|  |  | RS | 2,129 | -3.04 (-7.34, 1.27) | 2,128 | -2.97 (-7.30, 1.36) |
|  | TCGV | FHS-Gen 3 | 893 | **-2.8 (-5.6, -0.1)** | 889 | -2.7 (-5.6, 0.2) |
|  | HV | FHS-Gen 3 | 893 | 0.03 (-0.06, 0.13) | 889 | 0.00 (-0.09, 0.10) |
|  |  | RS | 1,963 | -0.02 (-0.10, 0.05) | 1,962 | -0.02 (-0.09, 0.06) |
|  | WMHV^†^ | FHS-Gen 3 | 893 | 0.18 (-0.05, 0.41) | 889 | 0.13 (-0.10, 0.37) |
|  |  | RS | 2,129 | 0.09 (-0.02, 0.21) | 2,128 | 0.05 (-0.06, 0.17) |

NAFLD, Non-alcoholic fatty liver disease; TBV, Total brain volume; TGV, Total gray matter volume; TCGV, Total cortical gray matter volume; HV, Hippocampal volume; WMHV, White matter hyperintensities volume; FHS, Framingham Heart Study, RS, Rotterdam study; SHIP, Study of Health in Pomerania, LSM, Liver stiffness measure. 

Model 1 adjusted for age, age-squared, sex, total intracranial volume, ethnicity and time between NAFLD assessment and MRI. 

Model 2 additionally adjusted for visceral adipose tissue (NAFLD)/ Body mass index (fibrosis), prevalent hypertension and prevalent diabetes.

Values in bold indicate p-value <0.05.

Values may slightly differ from the figures due to rounding

^†^ Log transformed

|  |  | Model 1 |  |  |  |  | Model 2 |  |  |  |  |
| --- | --- | --- | --- | --- | --- | --- | --- | --- | --- | --- | --- |
|  |  | Heterogeneity |  |  |  |  | Heterogeneity |  |  |  |  |
| Outcome | No. of studies | Chi^2^ | p-value | I^2 ^(%) | β (95% CI) | p-value | Chi^2^ | p-value | I^2 ^(%) | β  (95% CI) | p-value |
| Total brain volume | 4 | 0.90 | 0.82 | 0 | **-5.1 (-6.8, -3.4)** | **<0.001** | 2.35 | 0.50 | 0 | **-3.5 (-5.4, -1.7)** | **<0.001** |
| Total gray matter volume | 4 | 3.22 | 0.36 | 7 | **-2.2 (-3.7, -0.7)** | **0.004** | 0.10 | 0.99 | 0 | **-1.9 (-3.4, -0.3)** | **0.02** |
| Total cortical gray matter volume | 3 | 0.97 | 0.61 | 0 | **-3.1 (-4.8, -1.4)** | **<0.001** | 0.10 | 0.95 | 0 | **-1.9 (-3.7, -0.01)** | **0.05** |
| Hippocampal volume | 4 | 1.38 | 0.71 | 0 | -0.02 (-0.05, 0.02) | 0.34 | 0.42 | 0.94 | 0 | -0.02 (-0.05, 0.02) | 0.29 |
| WMHV | 4 | 1.77 | 0.62 | 0 | **0. 07 (0.02, 0.12)** | **0.01** | 1.02 | 0.80 | 0 | 0.04 (-0.02, 0.10) | 0.19 |

**Supplementary Table 2:** Pooled estimates of the associations between NAFLD and brain MRI measures- random effects

NAFLD, Non-alcoholic fatty liver disease; WMHV, White matter hyperintensities volume. 

Model 1 adjusted for age, age-squared, sex, total intracranial volume and time between NAFLD assessment and MRI. 

Model 2 additionally adjusted for body fat, prevalent hypertension and prevalent diabetes.

I², heterogeneity index
Values in bold indicate p-value <0.05.

**Supplementary Table 3:** Pooled estimates of the associations of liver stiffness and fibrosis with brain MRI measures- random effects 

|  |  |  | Model 1 |  |  |  |  | Model 2 |  |  |  |  |
| --- | --- | --- | --- | --- | --- | --- | --- | --- | --- | --- | --- | --- |
|  |  |  | Heterogeneity |  |  |  |  | Heterogeneity |  |  |  |  |
|  | Outcome | No. of studies | Chi^2^ | p-value | I^2 ^(%) | Total effect (95% CI) | p-value | Chi^2^ | p-value | I^2 ^(%) | Total effect (95% CI) | p-value |
| Liver stiffness measure (continuous) | Total brain volume | 2 | 1.6 | 0.21 | 37 | **-4.0 (-7.6, -0.4)** | **0.03** | 1.57 | 0.21 | 36 | -2.8 (-6.5, 0.8) | 0.13 |
|  | Total gray matter volume | 2 | 0.57 | 0.45 | 0 | **-2.4 (-4.7, -0.1)** | **0.04** | 0.93 | 0.33 | 0 | -2.1 (-4.5, 0.4) | 0.09 |
|  | Total cortical gray matter volume | 1 | N/A | N/A | N/A | -1.6 (-4.6, 1.4) | 0.30 | N/A | N/A | N/A | -1.4 (-4.4, 1.6) | 0.36 |
|  | Hippocampal volume | 2 | 4.4 | 0.04 | 77 | 0.04 (-0.1, 0.2) | 0.54 | 2.09 | 0.15 | 52 | 0.03 (-0.06, 0.11) | 0.53 |
|  | WMHV | 2 | 0.09 | 0.77 | 0 | 0.08 (-0.02, 0.18) | 0.13 | 0.01 | 0.94 | 0 | 0.03 (-0.07, 0.13) | 0.54 |
| Liver fibrosis (LSM ≥8.2 vs. <8.2 kPa) | Total brain volume | 2 | 3.32 | 0.07 | 70 | **-9.3 (-16.2, -2.3)** | **0.009** | 3.21 | 0.07 | 69 | **-7.9 (-14.8, -1.0))** | **0.03** |
|  | Total gray matter volume | 2 | 6.55 | 0.01 | 85 | -4.4 (-13.0, 4.2) | 0.32 | 7.21 | 0.007 | 86 | -4.1 (-13.4, 5.2) | 0.39 |
|  | Total cortical gray matter volume | 1 | N/A | N/A | N/A | -0.7 (-4.4, 3.0) | 0.71 | N/A | N/A | N/A | -0.3 (-4.1, 3.5) | 0.88 |
|  | Hippocampal volume | 2 | 2.27 | 0.13 | 56 | 0.04 (-0.08, 0.15) | 0.56 | 1.16 | 0.28 | 14 | 0.03 (-0.06, 0.11) | 0.57 |
|  | WMHV | 2 | 0.05 | 0.82 | 0 | 0.09 (-0.04, 0.23) | 0.18 | 0.08 | 0.78 | 0 | 0.04 (-0.10, 0.18) | 0.59 |
| Liver fibrosis (LSM ≥7.0 vs. <7.0 kPa) | Total brain volume | 2 | 0.97 | 0.32 | 0 | **-6.3 (-9.1, -3.5)** | **<0.001** | 0.93 | 0.34 | 0 | **-5.2 (-8.0, -2.4)** | **<0.001** |
|  | Total gray matter volume | 2 | 0.12 | 0.73 | 0 | **-2.4 (-4.7, -0.02)** | **0.05** | 0.21 | 0.65 | 0 | -2.2 (-4.5, 0.2) | 0.07 |
|  | Total cortical gray matter volume | 1 | N/A | N/A | N/A | **-2.8 (-5.6, 0.0)** | **0.05** | N/A | N/A | N/A | -2.7 (-5.6, 0.2) | 0.07 |
|  | Hippocampal volume | 2 | 0.66 | 0.42 | 0 | 0.0 (-0.06, 0.06) | 0.95 | 0.12 | 0.73 | 0 | -0.01 (-0.07, 0.04) | 0.66 |
|  | WMHV | 2 | 0.48 | 0.49 | 0 | **0.11 (0.01, 0.21)** | **0.03** | 0.38 | 0.54 | 0 | 0.06 (-0.03, 0.16) | 0.20 |

LSM, Liver stiffness measure; WMHV, White matter hyperintensities volume. 

Model 1 adjusted for age, age-squared, sex, total intracranial volume and time between fibrosis assessment and MRI. 

Model 2 additionally adjusted for body fat, prevalent hypertension and prevalent diabetes.

I², heterogeneity index
Values in bold indicate p-value <0.05.
