# Supplementary material for: Nonalcoholic fatty liver disease, liver fibrosis, and structural brain imaging: The Cross-Cohort Collaboration
Source: Eur J Neurol. Author manuscript; Available in PMC 2024 Feb 5. (PMC10840827; doi:10.1111/ene.16048)
Supplement: Supplementary file 2 [file NIHMS1931502-supplement-Supplement_2_Liver.docx]

**General information on participating studies**

**The Framingham Heart Study (FHS)** 

*General information*

The FHS is a community-based, prospective cohort study that was initiated in 1948 in the town of Framingham, Massachusetts, USA. The primary aim was to identify determinants of cardiovascular diseases and promote public health prevention.^1^ To date, three generations of participants have been recruited. Participants of the Original Cohort (N=5,209) began follow-up in 1948 ^1^. In 1971, follow-up was initiated on the Offspring cohort (N=5,124), which included the Original cohort’s Offspring and spouses of the Offspring.^2^ In 2002, individuals who had at least one parent in the Offspring cohort were recruited as part of the Third Generation cohort (Gen 3; N=4,095).^3^ Participants of the Offspring and 3rd Generation are follow approximately every 4 years. During the examination visit, a broad range of clinical, laboratory and imaging data are collected. Written informed consent is obtained at every visit from all participants. The study was approved by the institutional review board of Boston University Medical Center.

*Current analytical sample*

**NAFLD and brain MRI:** Data from the Offspring examination cycle 9 (N=2,430; 2011-2014) and the 3rd Generation examination cycle 2 (N=3,411; 2008-2011) were utilized for the current analysis. Of the total sample of 5,841 participants, 2,655 had information on NAFLD (1,244 Offspring and 1,411 Gen 3) and of them, 2,399 were aged ≥45 years (1,243 Offspring and 1,156 Gen 3). Of them, 1,615 (799 Offspring and 816 Gen 3) had information on brain MRI measures. After excluding 104 participants with prevalent stroke, dementia or other neurological disorders, another 395 participants with significant alcohol consumption (265 Offspring and 130 Gen 3) and additional 76 Gen 3 participants who used steatogenic medications, the final sample included 1,040 individuals (463 Offspring and 577 Gen 3).

**Liver stiffness and brain MRI:** Liver stiffness was measured in participants from the 3rd Generation at examination cycle 3 (N=3,171; 2016-2019). Of them, 3,077 underwent Fibroscan examination, all with valid results, and 1,203 additionally had information on brain MRI.  After exclusion of 64 individuals with prevalent dementia, stroke or other neurological disorder, another 77 who consumed excessive amount of alcohol drinks and additional 169 who used steatogenic medications, the final sample for this analysis included 893 participants.

*NAFLD assessment*

Multi-detector CT was performed using 8-slice MDCT technology (LightSpeed Ultra, General Electric, Milwaukee, WI). A calibration phantom (Image Analysis, Lexington, KY, USA) with a water equivalent compound (CT-Water, Light Speed Ultra, General Electric, Milwaukee, WI, USA) and calcium hydroxyapatite at 0, 75, and 150 mg/cm3 was placed under each participant.^4^ Three areas from the liver and one from an external phantom were measured, and the average of the liver measures were then calculated and used to create liver/phantom ratios. NAFLD was defined as having a liver/phantom ratio ≤0.33, consistent with prior FHS publications.^5^ Additional details on Multi-detector CT scan protocol and measurement of fatty liver can be found elsewhere ^5^. For the current analyses, we used NAFLD assessed between 2011-2014 in the Offspring and 2008-2011 in the 3rd Gen participants.

*Liver fibrosis assessment*

Liver fibrosis was assessed between 2016 and 2019 using a vibration-controlled transient elastography (VCTE) after at least 3 hours fasting. Participants were placed in the supine position with the right arm in maximal abduction and the skin exposed in the right upper quadrant. The VCTE probe was positioned in the intercostal space over the right lobe of the liver. Examiners applied the M probe first, but switched to the XL probe if needed based on the recommendations of the device and the manufacturer’s instructions. A minimum of 10 measurements were obtained from each participant, and median CAP and LSM values as well as their interquartile range were calculated by the device. Results were assessed for quality by a qualified hepatologist (M.T.L.). 

*Brain imaging*

MRI was done on a 1.5-Tesla Siemens Avanto scanner. Three-dimensional T1 and double echo proton density and T2 coronal images were acquired in 4-mm contiguous slices. All images were transferred to the centralized reading center at the University of California–Davis Medical Center and analyses were performed on QUANTA 6.2, a custom-designed image analysis package. Images were read centrally, blind to the subject’s identity, risk factors, and abdomen CT findings. Brain volume calculation was performed using semiautomated analysis of pixel distributions based on mathematical modeling of MRI pixel intensity histograms for cerebrospinal fluid (CSF) and brain matter (white matter and gray matter) to determine the optimal threshold of pixel intensity to best distinguish CSF from brain matter. Hippocampal volume was defined by the operator using automated traced boundaries. Total-, gray-matter, hippocampal and WMH volumes were adjusted for total cranial volume to correct for head size. Further details have been described previously.^6, 7^

*Study covariates*

Data collection including medical history, lifestyle and blood biomarkers was done during examination cycles through questionnaires and physical examinations. Type 2 diabetes was defined as a fasting serum glucose level ≥7.0 mmol/L (126 mg/dL), a non-fasting serum glucose level ≥11.1 mmol/L (200 mg/dL), and/or the use of blood glucose-lowering medication. Volume of visceral adipose tissue was assessed using a 8-slice supine multidetec- torCTas previously described.^8^ Hypertension was defined as systolic blood pressure ≥ 140 mmHg, diastolic blood pressure ≥ 90 mmHg or the use of antihypertensive medication.

*Funding*

This study was supported by grants from the National Heart, Lung, and Blood Institute contract for the Framingham Heart Study (contract No. N01-HC-25195, No. HHSN268201500001I, and No. 75N92019D00031), the National Institute on Aging (R01 AG054076, R01 AG049607, U01 AG052409, R01 AG059421, RF1 AG063507, RF1 AG066524, U01 AG058589) and the National Institute of Neurological Disorders and Stroke (R01 NS017950 and UH2 NS100605). 

**Rotterdam Study (RS)**

*General information*

The Rotterdam Study is a large, prospective, population-based cohort of the Netherlands including community-dwelling adults from Ommoord (a suburb of Rotterdam). A comprehensive description of the study design has been published previously.^9^ In summary, all inhabitants of Ommoord aged ≥55 years were invited to participate in 1990, from which 7983 individuals enrolled (RS-I). Subsequently, the cohort was expanded thrice: first in 2000, which resulted in the inclusion of 3,011 individuals who had reached the eligible age or had moved into the study area (RS-II), later in 2006, with 3,932 individuals aged 45 or over (RS-III) and recently in 2015 with 3,005 individuals aged 40 or over (RS-IV). Participants take part in comprehensive interviews and visit the research facility for an in-person examination every 3-6 years. In addition, participants are monitored continuously through electronic linkage of medical records with the study database. 

The Rotterdam Study has been approved by the Medical Ethics Committee of the Erasmus University Medical Center (registration number MEC 02.1015), in accordance with the Population Screening Act, which is executed by the Dutch Ministry of Health, Welfare and Sport (license number 1071272-159531-PG).

*Current analytical sample*

The current study comprised 2,826 participants who underwent abdominal ultrasound for the assessment of NAFLD between 2009 and 2014. A subsample of these participants (N = 2,129) also underwent liver stiffness measurement (LSM) to assess fibrosis.

*NAFLD assessment*

Steatosis based on **abdominal ultrasound** was defined as hyperechoic liver parenchyma compared to the spleen or kidney according to the protocol of Hamaguchi et al.^10^ Abdominal ultrasound was performed by a single certified and experienced sonographer (PVW) on a Hitachi Hi Vision 900.

*Liver fibrosis assessment*

Liver stiffness was assessed using transient elastography (FibroScan, EchoSens, Paris, France). At least 10 measurements were obtained through either M or XL probe according to the device's instructions. Final measurements >7.1 kPa with an interquartile range >30% were considered unreliable and discarded.^11^ Liver fibrosis was defined as liver stiffness measurement (LSM) ≥ 8.2 kPa or ≥ 7.0 kPa.^12^ 

*Brain imaging*

MRI of the brain was performed on a 1.5T scanner (General Electric Healthcare, Milwaukee, WI) using an 8-channel head coil. Imaging acquisition included a high-resolution axial T1-weighted sequence, a fluid-attenuated inversion recovery sequence, a proton density–weighted sequence, and a T2*-weighted gradient echo sequence. Details about the sequences, preprocessing, and the classification algorithm have been described previously.^13^ Total intracranial and tissue volumes and volume of white matter hyperintensities (WMHs) were quantified via automated tissue segmentation.^14^ These segmentations were visually inspected and manually corrected if needed. Segmentation of the hippocampus was performed using FreeSurfer 6.1.^15^

*Study covariates*

Medical history and socio-demographic variables were assessed by standardized questionnaires during home interview. Blood samples, and measurement of body weight and height were carried out during routine examinations at the research center. Body mass index was calculated with weight and height (kg/m^2^, calculated by weight [kg] divided by height [m] squared). Type 2 diabetes was defined as a fasting glucose ≥7.0mmol/L, non-fasting glucose ≥11.1mmol/L, the use of oral antidiabetic medication, or use of Insulin. Hypertension was defined as systolic blood pressure ≥ 140 mmHg, diastolic blood pressure ≥ 90 mmHg or the use of antihypertensive medication.

*Funding*

The Rotterdam Study is supported by the Erasmus MC University Medical Center and Erasmus University Rotterdam, the Netherlands Organization for Scientific Research (NWO), the Netherlands Organization for Health Research and Development (ZonMW), the Research Institute for Diseases in the Elderly (RIDE), the Ministry of Education, Culture and Science, the Ministry of Health, Welfare and Sport, The European Commission (DGXII), the Netherlands Genomics Initiative (NGI), and the Municipality of Rotterdam. This study was partly performed as part of the Netherlands Consortium of Dementia Cohorts (NCDC), which receives funding in the context of Deltaplan Dementie from ZonMW Memorabel (projectnr 73305095005) and Alzheimer Nederland.

**Study of Health in Pomerania (SHIP)**

The Study of Health in Pomerania (SHIP) is a prospective population-based cohort study.^16, 17^ It was established to examine the health and disease status of the general adult population of West Pomerania, a north-eastern region in Germany of approximately 220,000 inhabitants which had the lowest life expectancy in Germany in the early 1990s. It comprises the two independent cohorts SHIP-START (recruited between 1997 and 2001) and SHIP-TREND (recruited between 2008 to 2012). From the total population of West Pomerania, a two-stage stratified cluster sample of 7,008 adults aged 20–79 years was drawn and 4,308 subjects agreed to participate in the baseline examination (SHIP-START-0). A separate stratified random sample of 8,016 adults aged 20–79 years was drawn for SHIP-TREND. The target sample size was chosen to obtain a final sample size similar to that of SHIP-START-0. 4,420 subjects agreed to participate in the baseline examinations (SHIP-START-0). All participants gave written informed consent. The study was approved by the ethics committee of the University Medicine Greifswald and complies with the declaration of Helsinki. The second follow-up of SHIP-START (SHIP-START-2) and baseline SHIP-TREND-0 were the first studies worldwide to utilize whole-body magnetic resonance imaging (MRI) in a general population setting.^18^

*Current analytical sample*

The data used in our analyses were derived from the second follow-up examinations of SHIP-START and the baseline examinations of SHIP-TREND, which were conducted between 2008 and 2012. Data on NAFLD and brain MR images were available for 2,300 participants. 92 participants were excluded because of major structural abnormalities of the brain (e.g. large cysts, brain tumors), prevalent stroke, or dementia. Additionally, 414 participants who had significant alcohol consumption (women: >12g/d, men: >24g/d during last 30 days) or took steatogenic medication were excluded. The final analytic sample comprised data of 1,794 participants.

*NAFLD assessment*

Liver ultrasound examination was performed between the years 2008 and 2012 by examiners using a transportable B-mode ultrasound device (Vivid I; GE-Healthcare, Waukesha, WI, USA) with a 2.5 MHz ultrasonic transducer. A 2-point scale was used to assess the presence of hepatic steatosis: (0) no steatosis, and (1) steatosis. Hepatic steatosis was defined as a hyperechogenic liver pattern in comparison to the renal cortex.^19^

*Brain imaging*

T1-weighted and fluid-attenuated inversion recovery (FLAIR) scans of the head were obtained with a 1.5 T Siemens Magnetom Avanto scanner (Siemens, Erlangen, Germany).^18^ The following parameters were used: T1: orientation=axial plane, TR=1,900 ms, TE=3.37 ms, flip angle 15 °, slice thickness=1 mm, and resolution 1 mm x 1 mm, FLAIR: orientation=axial plane, TR=5,000 ms, TE=325 ms, slice thickness=3 mm, and resolution 0.9 mm × 0.9 mm.

T1-weighted scans were processed with the image-processing pipeline FreeSurfer version 7.1, which is documented and freely available for download online ([http://surfer.nmr.mgh.harvard.edu](http://surfer.nmr.mgh.harvard.edu/)).^20^ The processing includes segmentation of the cerebral cortex and subcortical regions as well as calculation of total brain volume, gray matter volume, and hippocampal volume (sum of left and right hippocampal volume). FreeSurfer also gives an estimate of the total intracranial volume, which can be used to account for some of the variability between the study participants. After preprocessing and coregistration of T1-weighted and FLAIR scans, white matter lesions were segmented using the Brain Intensity AbNormality Classification Algorithm.^21^ 

*Study covariates*

Medical history and socio-demographic variables were assessed by standardized questionnaires during a computer-assisted face-to-face interview. Blood samples, and measurement of body weight and height were carried out during subsequent medical examinations. Body mass index was calculated by dividing weight [kg] by height [m] squared. Diabetes was defined either based on self-report, intake of anti-diabetic medication (ATC code A10), glycated hemoglobin ≥ 6.5% (International Expert Committee 2009), or blood glucose ≥ 11.1 mmol/l (IDF-WHO 2006). Hypertension was defined as systolic blood pressure ≥ 140 mmHg, diastolic blood pressure ≥ 90 mmHg or intake of antihypertensive medication. Blood pressure was measured three times and the average of the second and third measurement was considered.

*Funding*

The Study of Health in Pomerania (SHIP) is part of the Community Medicine Research net (CMR) (http://www.medizin.uni-greifswald.de/icm) of the University of Greifswald funded by grants from the German Federal Ministry of Education and Research (BMBF, grant 01ZZ96030, 01ZZ0701). The MRIs in SHIP were supported by a joint grant from Siemens Healthineers, Erlangen, Germany, and the Federal State of Mecklenburg-Western Pomerania. This study was further supported by National Institute of Health (NIH) grant AG059421.

*Acknowledgments*

The Study of Health in Pomerania (SHIP) is part of the Community Medicine Research net (CMR) (http://www.medizin.uni-greifswald.de/icm) of the University Medicine Greifswald, which is supported by the German Federal State of Mecklenburg- West Pomerania. MRI scans in SHIP and SHIP-TREND have been supported by a joint grant from Siemens Healthineers, Erlangen, Germany and the Federal State of Mecklenburg-West Pomerania. 

1.	Dawber TR, Kannel WB. The Framingham study. An epidemiological approach to coronary heart disease. *Circulation* 1966;34(4):553-555 doi: 10.1161/01.cir.34.4.553.

2.	Feinleib M, Kannel WB, Garrison RJ, McNamara PM, Castelli WP. The Framingham Offspring Study. Design and preliminary data. *Prev Med* 1975;4(4):518-525 doi: 10.1016/0091-7435(75)90037-7.

3.	Splansky GL, Corey D, Yang Q, et al. The Third Generation Cohort of the National Heart, Lung, and Blood Institute's Framingham Heart Study: design, recruitment, and initial examination. *Am J Epidemiol* 2007;165(11):1328-1335 doi: 10.1093/aje/kwm021.

4.	Speliotes EK, Massaro JM, Hoffmann U, et al. Liver fat is reproducibly measured using computed tomography in the Framingham Heart Study. *J Gastroenterol Hepatol* 2008;23(6):894-899 doi: 10.1111/j.1440-1746.2008.05420.x.

5.	Speliotes EK, Massaro JM, Hoffmann U, et al. Fatty liver is associated with dyslipidemia and dysglycemia independent of visceral fat: the Framingham Heart Study. *Hepatology* 2010;51(6):1979-1987 doi: 10.1002/hep.23593.

6.	DeCarli C, Massaro J, Harvey D, et al. Measures of brain morphology and infarction in the framingham heart study: establishing what is normal. *Neurobiol Aging* 2005;26(4):491-510 doi: 10.1016/j.neurobiolaging.2004.05.004.

7.	Jeerakathil T, Wolf PA, Beiser A, et al. Stroke risk profile predicts white matter hyperintensity volume: the Framingham Study. *Stroke* 2004;35(8):1857-1861 doi: 10.1161/01.STR.0000135226.53499.85.

8.	Fox CS, Massaro JM, Hoffmann U, et al. Abdominal visceral and subcutaneous adipose tissue compartments: association with metabolic risk factors in the Framingham Heart Study. *Circulation* 2007;116(1):39-48 doi: 10.1161/circulationaha.106.675355.

9.	Ikram MA, Brusselle G, Ghanbari M, et al. Objectives, design and main findings until 2020 from the Rotterdam Study. *Eur J Epidemiol* 2020;35(5):483-517 doi: 10.1007/s10654-020-00640-5.

10.	Hamaguchi M, Kojima T, Itoh Y, et al. The severity of ultrasonographic findings in nonalcoholic fatty liver disease reflects the metabolic syndrome and visceral fat accumulation. *Am J Gastroenterol* 2007;102(12):2708-2715 doi: 10.1111/j.1572-0241.2007.01526.x.

11.	Boursier J, Zarski JP, de Ledinghen V, et al. Determination of reliability criteria for liver stiffness evaluation by transient elastography. *Hepatology* 2013;57(3):1182-1191 doi: 10.1002/hep.25993.

12.	Roulot D, Costes JL, Buyck JF, et al. Transient elastography as a screening tool for liver fibrosis and cirrhosis in a community-based population aged over 45 years. *Gut* 2011;60(7):977-984 doi: 10.1136/gut.2010.221382.

13.	Ikram MA, van der Lugt A, Niessen WJ, et al. The Rotterdam Scan Study: design update 2016 and main findings. *Eur J Epidemiol* 2015;30(12):1299-1315 doi: 10.1007/s10654-015-0105-7.

14.	de Boer R, Vrooman HA, van der Lijn F, et al. White matter lesion extension to automatic brain tissue segmentation on MRI. *Neuroimage* 2009;45(4):1151-1161 doi: 10.1016/j.neuroimage.2009.01.011.

15.	Schmidt MF, Storrs JM, Freeman KB, et al. A comparison of manual tracing and FreeSurfer for estimating hippocampal volume over the adult lifespan. *Hum Brain Mapp* 2018;39(6):2500-2513 doi: 10.1002/hbm.24017.

16.	Völzke H, Alte D, Schmidt CO, et al. Cohort profile: the study of health in Pomerania. *International journal of epidemiology* 2011;40(2):294-307.

17.	Völzke H, Schössow J, Schmidt CO, et al. Cohort profile update: the Study of Health in Pomerania (SHIP). *International journal of epidemiology* 2022;51(6):e372-e383.

18.	Hosten N, Bülow R, Völzke H, et al. SHIP-MR and radiology: 12 years of whole-body magnetic resonance imaging in a single center.  Healthcare; 2022: Multidisciplinary Digital Publishing Institute: 33.

19.	Naeem M, Markus MR, Mousa M, et al. Associations of liver volume and other markers of hepatic steatosis with all‐cause mortality in the general population. *Liver International* 2022;42(3):575-584.

20.	Fischl B. FreeSurfer. *Neuroimage* 2012;62(2):774-781.

21.	Griffanti L, Zamboni G, Khan A, et al. BIANCA (Brain Intensity AbNormality Classification Algorithm): A new tool for automated segmentation of white matter hyperintensities. *Neuroimage* 2016;141:191-205.
